# Exploring Perceptions of Control Within Offender Cognition and Recidivism Paradigms

**DOI:** 10.5964/ejop.5997

**Published:** 2022-11-30

**Authors:** Anistasha H. Lightning, Danielle Polage

**Affiliations:** 1 Department of Psychology, Central Washington University, Ellensburg, WA, USA; California University of Pennsylvania, California, PA, USA

**Keywords:** control, helplessness, incarceration, recidivism, rehabilitation

## Abstract

Elements of perceived control are associated with recidivism in offender populations. We investigated the application of locus of control to the frequency of personal involvement with the law and to beliefs surrounding the likelihood of future contact with the legal system. We hypothesized that, as the number of sentencings or legal experiences increased, locus of control would externalize. We also predicted that increased legal involvement would lead to greater belief in the likelihood of future involvement. A statistically significant path model suggests that locus of control appears to be a predictor of increased criminality, as opposed to the other way around. Further, data suggests that an offender will view future legal involvement as more likely if they have experienced greater lifetime contact with the legal system. We speculate on the possible application of these data to intervention strategies identifying offenders with high priority intervention needs.

Consistent stress, loss of control, and hopelessness have been linked to recidivism and external locus of control in both adult and juvenile populations (Barnett & Fitzalan Howard, 2018; Halliday & Graham, 2000; Nowicki et al., 2018). Locus of control, a construct first described by Rotter (1966), appears to be an influential factor in offender rehabilitation and recidivism reduction. Internalizing locus of control, or the belief that one has control over what happens to them, is associated with improvements in mental health pathologies, drug addiction, general treatment amenability, decreases in future criminality for both adults and juveniles, and increased personal wellbeing in offender populations (Asberg & Renk, 2014; Morgan & Flora, 2002; Page & Scalora, 2004; van der Helm et al., 2009).

Unfortunately, many offenders enter the legal system with more externalized locus of control, the belief that they have little or no control over what happens to them. Understanding more about where this externalization comes from may help correctional systems to better rehabilitate offenders toward more internalized control thinking.

Many express hopeless resignations when faced with the prospect of incarceration for current or continued criminal activity. Often, offenders attribute criminality to survival necessities and to a sense of resignation directed at their surrounding environments (Halliday & Graham, 2000). This application of helpless thinking is tied to mental and emotional rehabilitative outcomes in both adult and juvenile populations. Feelings of control, helplessness, and resignation have been linked to low levels of treatment amenability, increased lifelong criminality, depression, and lower levels of adaptive resilience (Archer, 1980; Chorpita & Barlow, 1998; Contreras et al., 2011; Han et al., 2001; Munoz et al., 2017; Page & Scalora, 2004).

These feelings can be perpetuated by consistent exposure to stressful external factors (Nowicki et al., 2018; Takase et al., 2005). These can have a significant influence on criminality. Family setting factors such as broken homes, low incomes, and deprived neighborhoods are linked to recidivism in juvenile offenders (Contreras et al., 2011). In adults, a lack of rehabilitative efforts tied to emotional and economic health may be predictive of recidivism (Gaum et al., 2006).

Consistent and seemingly inescapable stress, such as relationship stress, factors of poverty, violence exposure, and drug use are strongly associated with criminality. These stressors may influence the development of external locus of control (Abrams & Snyder, 2010; Lodewijks et al., 2010; Nowicki et al., 2018; Passini, 2012; Richaud, 2013). This is often expressed behaviorally as hopelessness, and may be an important influencing factor in recidivism, or the tendency to relapse into criminal behavior after incarceration or other legal intervention (Halliday & Graham, 2000; Takase et al., 2005).

This hopelessness may lead individuals back toward criminality if their surrounding environmental factors do not change. Up to 83% of offenders in the United States are re-arrested or re-incarcerated within three years of their release from detention (U.S. Department of Justice, 2018). This can reinforce feelings of futility and hopelessness, antecedents to external locus of control (Halliday & Graham, 2000; Nowicki et al., 2018; Takase et al., 2005).

It may thus be possible to use locus of control measurements to identify those individuals at greater need for intervention early on in their involvement with the legal system. In order to do this, however, the predictive relationship between the two must first be demonstrated empirically. This is the goal of the present study.

## Perceptions of Control and Antecedents to Recidivism

Factors such as poverty, dangerous living conditions, and social influences are often linked to criminal behavior (Abrams & Snyder, 2010; Halliday & Graham, 2000; Passini, 2012). These factors can lead to significant exposure stress even outside of the correctional system, removal of feelings of control, and may affect resiliency. All of these have been identified as potential antecedents to recidivism (Han et al., 2001; Lodewijks et al., 2010; Richaud, 2013).

Locus of control does not appear to be a static construct. Rather, the literature points to a more dynamic and malleable nature where locus of control may change and shift from internal to external and back again based on varying environmental factors (Nowicki et al., 2018). This is flexibility is particularly emphasized in changing social relationships and through the stability of surrounding environments (Kubala et al., 2012; Maier, 2001; Nowicki et al., 2018; Takase et al., 2005). Thus, sustained exposure to social and environmental stressors is a predictor of an externalizing locus of control over time (Nowicki et al., 2018).

This principle of flexibility is an important one for the investigation of externalizing locus of control and its potential use as an early indicator of intervention need. If locus of control can be changed based on environmental factors and treatment, then it may be possible to use the construct as a viable testing platform for intervention programs.

Concerning the potential applications to rehabilitative practice, internal locus of control has been linked to adaptive behaviors and high levels of resilience (Munoz et al., 2017). External locus of control has been linked to depression, anxiety, and other psychopathologies (Archer, 1980; Chorpita & Barlow, 1998; Han et al., 2001). Since locus of control appears to be elastic, the link to both adaptive and maladaptive mental formations is important for its potential as an early intervention and treatment amenability tool (Archer, 1980; Chorpita & Barlow, 1998; Han et al., 2001; Munoz et al., 2017; Nowicki et al., 2018; Page & Scalora, 2004).

The increased stress exposure, autonomy restriction, social restriction, and danger exposure often coupled with incarceration may, then, influence the development of the more maladaptive external locus of control (Dmitrieva et al., 2012; Halliday & Graham, 2000; Nowicki et al., 2018; Takase et al., 2005). Internal locus of control is far more psychologically adaptive, with links to increased resiliency, positive behavior patterns, and better stress management, several of which are linked to better behavioral control (Dmitrieva et al., 2012; Munoz et al., 2017).

This has significant implications for treatment efficacy in offender populations as well. Any intervention focused on controlling the individual, deterrence, building extrinsic motivation, or basic discipline does little to curb recidivism. This is true for interventions based inside a correctional facility or out in the community. However, programs focused on building intrinsic motivation and those based upon restorative intent are far more effective in reducing recidivism (Barnett & Fitzalan Howard, 2018).

This is particularly impactful here because of the analytic distinction between extrinsic ineffectiveness and intrinsic effectiveness. Extrinsic interventions, or those designed to emphasize external reasoning, appear to be ineffective in dealing with recidivism while those focused on building internal motivation for change and behavioral decisions, appear to be effective in curbing recidivism (Barnett & Fitzalan Howard, 2018). Therefore, those with higher individual recidivism rates may have a greater degree of external reasoning which may reflect external locus of control.

## Behavioral Disturbance, Locus of Control, and Recidivism

Incarceration itself results in behavioral disturbances and significant mental health consequences that may lead to repeat incarceration, particularly in juvenile offenders (Lambie & Randell, 2013). Indeed, criminal behavior continues from adolescence to adulthood in as many as 40% to 60% of offenders in the United States (National Institute of Justice, 2013).

More internal orientations predict greater levels of help-seeking behavior, treatment participation, and positive treatment outcomes. Individuals with more external orientation displayed more resistance to behavioral changes and treatment strategies. These are significant locus of control-based influencers on treatment amenability (Page & Scalora, 2004).

Helplessness is a related concept when discussing treatment and intervention efficacy. It is a depression-like behavior observed consistently in animals and humans when perceived control over the environment is lacking (Kubala et al., 2012; Maier, 2001; Takase et al., 2005). This feeling can be reinforced by repeated and failed attempts to escape from the adverse environment and has links to externalized locus of control (Cohen et al., 1976; Hiroto, 1974; Takase et al., 2005).

This construct has a neurobiological foundation, granting important biological validity to this investigation. Repeated exposure to inescapable tail shocks in rats results in decreased observable desire to leave the aversive environment, decreases social exploration, and activates the serotonergic dorsal raphe nucleus, an area of the brain implicated in depression (Kubala et al., 2012; Takase et al., 2005). This type of escape-avoidance has been linked to learned helplessness in humans, which itself has been previously linked to external locus of control, depression, anxiety, and other pathologies (Archer, 1980; Chorpita & Barlow, 1998; Cohen et al., 1976; Han et al., 2001; Hiroto, 1974; Maier, 2001).

For offenders, failed attempts to break a criminal cycle or feelings of hopelessness relating to uncontrollable environmental factors create stressors that influence criminal continuity (Halliday & Graham, 2000; Page & Scalora, 2004). Feelings of control, treatment amenability, and stress exposure have been identified as related environmental factors with ties to locus of control (Barnett & Fitzalan-Howard, 2018; Halliday & Graham, 2000; Page & Scalora, 2004).

## The Current Study

Given established relationships between locus of control, environmental factors, treatment amenability, and lifetime recidivism risk, we examined whether repeated contact with the legal system is related to externalizing locus of control in adult criminal offenders. From the available literature, we developed and tested the following hypothesis: as the number of sentencings or legal experiences increases, locus of control will predictively externalize. Meaning that the more times an individual has been in contact with the legal system, the more they will view the causes of their legal involvement as external. We further hypothesized that increased legal involvement would lead to greater belief in the likelihood of future legal involvement.

## Method

### Procedure

This research utilized a correlational design. All participants were given the same surveys in similar conditions. Informed consent was obtained from each participant, and all participants were educated on the definitions of the variables before completing the survey to ensure self-reporting could be as accurate as possible.

Surveys were administered on a volunteer, one-on-one basis in designated jail visitation areas or in the inmate’s personal living area. Participants retained the option to fill out the survey on their own or have the administrator read the survey to them and transcribe their responses to compensate for any limited literacy, educational disadvantage, or other written language barriers.

### Participants

We surveyed 117 county jail inmates in Washington, a state in the northwest of the United States. In order to participate in the study, volunteers needed to be currently incarcerated in a county jail and at least 18 years of age. Additionally, they needed to understand written or spoken English at an appropriate level to receive informed consent, as we did not have the ability to provide translators for non-English speaking participants. Five surveys were removed from analysis for lack of completion or as extreme outliers. The final sample for analysis was *N =* 112.

### Materials

#### Demographics

In order to ensure the protection of inmate identity, minimal demographic data were collected, at the insistence of both the participating jail facilities and the Institutional Review Board (IRB). This was due to the potentially undecided nature of some legal cases, as county jail inmates may be awaiting trial, sentencing, or some part of their record may be sealed. This was an unfortunate omission, as we lost any potential ability to evaluate relationships within these demographic areas. Nonetheless, compliance with this requirement was necessary for the completion of the research.

Participants were asked to answer limited demographic questions regarding their age, race/ethnicity, education level, household type (to determine homelessness status), and household income. It is important to note that demographic factors were recorded only to give a descriptive representation of the population. Excluding age, which we did analyze in line with established age-crime relationships, they were otherwise not intended to be used in the analysis and were not expressly relevant to the hypothesis. Additionally, many participants did not know their household income, and thus we lacked significant information to use this variable in the analysis.

Of course, we understand that there may be significant relationships between these factors and locus of control and are aware that these factors play important parts as antecedents to criminality. However, our hypothesis sought to explore only the relationships between locus of control, legal involvement, and predictive thoughts on future incarceration. A detailed analysis on the relationship between these variables and our demographic identifiers is both interesting and valuable, but beyond the intended scope of this research.

#### Involvement With the Law Questionnaire

We developed a questionnaire designed to record self-reported data concerning the legal history of study participants. This questionnaire asked participants to estimate their lifetime “legal involvement”, defined as the number of times they had experienced arrest, jail time awaiting trial or court date, jail sentences, prison sentences, electronic home monitoring (house arrest), community service or community restitution, group homes or work release programs, probation, and “other” involvement with the law, which they were asked to explain. We computed “legal involvement” as the sum-total of all self-reported involvement in these categories. To develop the list of categories, we consulted with participating jail facilities and a corrections expert present on the IRB in order to determine possible sentencing types.

Participants then used a Likert-type scale to indicate how likely, if at all, they believed they were to be involved with the law post-release, which we termed “future likelihood”. Finally, they were presented with an open-ended question which prompted them to reflect on the causes for their current and past legal involvement. For the Involvement with the Law Questionnaire, Cronbach’s alpha was .78.

#### The Revised Causal Dimensions Scale (McAuley et al., 1992)

McAuley et al.’s (1992) Revised Causal Dimension Scale immediately followed the Involvement with the Law Questionnaire. It divides locus of control into four dimensions, namely “locus of causality,” “stability,” “personal control,” and “external control.” The scale was used with permission from the copyright owner, Sage Publications. The original scale authors performed reliability testing during development of the scale. The average internal consistencies (Cronbach’s alpha) across the four categories were .67 for locus of causality, .67 for stability, .79 for personal control, and .82 for external control (McAuley et al., 1992).

The Revised Causal Dimension Scale was selected because it is designed to examine locus of control with respect to prompting scenarios, allowing locus of control to be examined with specific respect to legal involvement (McAuley et al., 1992; Russell, 1982). This allowed us to examine locus of control with specific respect to thoughts on incarceration. This was a great boost to the ability to evaluate our specific hypothesis using validated study instruments developed for locus of control research. In our case, the prompting scenario was the open-ended question discussed above.

#### Data Coding Procedure

We coded data based on participant responses to numeric or scale-based questions. Some responses were converted to bivariate data to reflect affirmative/negative answer types. We recorded self-reported frequencies of juvenile legal involvement and lifetime legal involvement. We calculated a third variable, adult legal involvement, as the difference between lifetime and juvenile legal involvement. We were not able to access personal files to verify these numbers, as county jail inmates typically have protected files, and thus rely only on the assumed accuracy of the self-reported frequencies.

We coded two different variables concerning future likelihood. The first was a dichotomous response variable concerning whether participants thought future legal involvement was likely. Those that selected “yes” were asked to rate how likely future legal involvement was to occur on a Likert-type scale. A score of one denoted “extremely unlikely,” and a score of eight denoted “extremely likely.” A score of zero was coded for all participants who indicated “no” on the bivariate question, to indicate they did not believe there was any likelihood of future involvement.

Finally, we recorded total scores for each of the four Revised Causal Dimension Scale elements, termed “locus of causality,” “stability,” “personal control,” and “external control” after their original operational designations (McAuley et al., 1992). The scale is divided into 12 Likert-type questions, with response values ranging from 1 to 9. Each of the scale’s four metrics are measured by three of these questions. We computed total values for each of the four metrics as a sum of response values for each of these three sub-divided questions. The specific divisions used were established by the original scale authors.

## Results

We first performed a preliminary data check to examine our data for normality. Some data were highly kurtotic, and thus we performed appropriate logarithmic transformations to achieve the required normality for further parametric testing. We then performed descriptive analysis, correlational analysis and linear regression analysis to extrapolate meaning from the data using a combination of R, a programming language used for statistical analysis, IBM’s SPSS statistics software, and G*Power analysis software.

### Demographics

The mean age of participants was 33.73 years (*SD* = 8.886), 75.9% reported graduating from high school or a General Education Diploma (G.E.D.) program, and 33.9% reported being homeless before becoming incarcerated. Ethnically, the participants identified as White (48.2%, *n* = 54), Hispanic or Latin American (17.9%, *n* = 20), Black or African American (2.7%, *n* = 3), Native American or Alaska Native (9.8%, *n* = 11), Asian (1.8%, *n* = 2), Multiracial (15.2%, *n* = 17), or some “other” ethnicity (4.5%, *n* = 5).

### Power Analysis

We first performed a power analysis to determine the required sample size for a medium effect size and an acceptable statistical power factor of .85 at the *p* = .05 and *p* = .01 levels for Pearson’s correlations and linear regression, using G*Power version 3.1.9.2 for this analysis (Faul et al., 2007).

For Pearson’s *r*, analysis predicted that 163 participants would yield a .85 power factor at the *p* = .05 level, along with 255 participants at the *p* = .01 level. A medium effect size for a Pearson Correlation is approximately .30, according to the G*Power software we utilized (Faul et al., 2007). For linear regression analysis, the recommended *n* was 87 at the *p* = .05 level, given three predictor variables, 76 for two predictors, and 62 for one predictor. These numbers increase to 120 participants for three predictors, 108 for two, and 91 for one at the *p* = .01 level. For linear regression, a medium effect size is considered to be .15.

For linear regression analysis, our sample size effectively met size requirements for a .85 power factor. The regression analysis was the most important analysis for our hypothesis, so the effect size here is promising. However, our sample size did not meet the size requirement for a .85 power factor and medium effect size for Pearson’s *r* correlation. Nonetheless, data show statistically significant results. Additionally, we chose to perform correlational analysis in order to establish preliminary relationships between the data for reference in future studies of this type. Indeed, these relationships may be used as a baseline for future analysis on larger participant groups, which may increase the statistical power of the results. Thus, correlation analysis was maintained both to contribute to the future of scientific discussion on this topic and due to the presence of statistically significant results despite the lower statistical power.

### Descriptive Statistics

Concerning past legal involvement, 56.3% of participants (*n* = 112) reported a juvenile criminal record (*n* = 63). Concerning future legal involvement, 68.8% indicated that they believed future legal involvement was likely (*n* = 77). The mean future likelihood score across participants was 3.67 (*SD* = 3.08).

We divided legal involvement into juvenile, adult, and lifetime frequencies. The mean juvenile legal involvement was 11.47 individual instances of involvement (*SD* = 25.57). For adult and lifetime legal involvement, the mean frequencies were 51.19 (*SD* = 66.02) and 62.65 (*SD* = 77.18), respectively.

We divided lifetime legal involvement into low, moderate, and high levels of involvement. These are defined as total involvement greater than 0.5 standard deviations below the mean for low, within +/- 0.5 standard deviation from the mean for moderate, and greater than 0.5 standard deviation for high involvement. These parameters were selected in order to achieve the most even distribution possible between the categories. Maintaining a standard deviation difference of less than 1 likewise ensured that the low and high categories would represent more than just the extreme ends of the overall distribution, giving a more realistic picture of the differences between participants. The low legal involvement category represented only 28.6% of participants (*n* = 32), while the moderate and high categories represented 38.4% (*n* = 43) and 33.0% (*n* = 37), respectively.

For the Revised Causal Dimensions Scale, the range of response scores was between 3 and 27. For locus of causality, the mean score was 17.11 (*SD* = 6.00). External control had a mean score of 13.71 (*SD* = 6.69), while the mean stability score was 12.28 (*SD* = 5.82). Finally, personal control exhibited a mean score of 17.41 (*SD* = 6.20).

### Correlations

We first examined correlation coefficients between hypothesis-testing variables, the results of which are summarized in Table 1.

**Table 1:** Correlations and p-Values for Study Variables

Variable	1	2	3	4	5	6	7	8	9
1. Age	—								
2. Lifetime Legal Involvement	.195* (.040)	—							
3. Adult Legal Involvement	.225* (.017)	.950**(.001)	—						
4. Juvenile Legal Involvement	-.091 (.341)	.525** (.000)	.303** (.001)	—					
5. Future Likelihood	.057 (.552)	.352** (.000)	.334 (.000)	.236* (.012)	—				
6. Locus of Causality	.139 (.144)	.200* (.035)	.238* (.012)	.067 (.482)	.085 (.375)	—			
7. External Control	-.020 (.833)	-.024 (.798)	-.048 (.616)	.068 (.476)	.081 (.393)	-.314** (.064)	—		
8. Stability	.134 (.159)	.030 (.754)	-.036 (.706)	.201* (.033)	.252** (.007)	-.093 (.330)	.176 (.064)	—	
9. Personal Control	-.069 (.472)	-.054 (.575)	-.030 (.752)	-.034 (.721)	-.216* (.022)	.343** (.000)	-.316** (.001)	-.286** (.002)	—

*Note*. Table entries are Pearson's correlations for study variables with *p*-values in parentheses (*n* = 112)

*p < .05. **p < .01.

Future likelihood was statistically significantly correlated with juvenile, adult, and lifetime legal involvement, *r*(110) = .24, *p* = .01, *r*(110) = .33, *p* = .000, and *r*(110) = .35, *p* = .000, respectively. All correlations were positive, suggesting a positive relationship between these variables.

Future likelihood was significantly and positively correlated with the stability element in McAuley et al.’s (1992) Revised Causal Dimension Scale, *r*(110) = .25, *p* = .007. This reflects a relationship between the belief in future legal involvement and the perception of surrounding environmental stability. In our case, it is likely that “stability” reflects a continuity of the conditions that surrounded incarceration, thus influencing future likelihood predictions in a positive correlational relationship.

Locus of causality was significantly correlated with adult and lifetime legal involvement, *r*(110) = .24, *p* = .01; *r*(110) = .20, *p* = .04, but not with juvenile legal involvement, *r*(110) = .14, *p* = .15. Stability was significantly correlated with juvenile legal involvement, *r*(110) = .20, *p* = .03, and future likelihood, *r*(110) = .25, *p* = .01.

Personal control and external control were minimally correlated with legal involvement. Personal control was only statistically significantly correlated with future likelihood, *r*(110) = -.22, *p* = .02, and external control was not statistically significantly correlated with any of the causal dimensions variables.

Some correlations are present between the various Revised Causal Dimension Scale elements, all of which reflect correlations measured by the original scale authors. Our data showed a significant negative correlation between personal control and both external control and stability, *r*(110) = -.32, *p* = .001; *r*(110) = -.29, *p* = .002. There is a significant positive correlation between personal control and locus of causality, *r*(110) = .34, *p* = .000. These relationships reflect the statistically significant correlations between the same variables identified by the original authors at the *p* < .05 level.

### Regression Models

**Figure 1:**
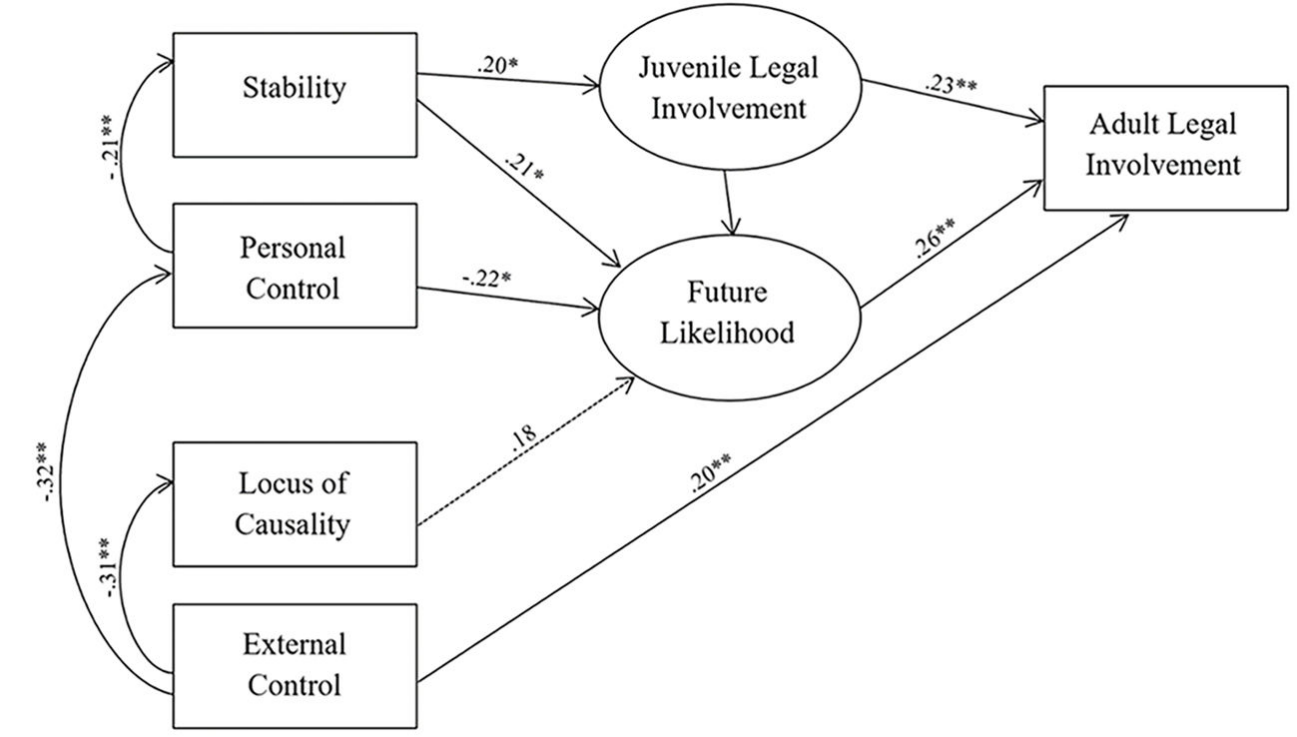
Path Analysis Model of Associations Between Locus of Control Elements and Legal Involvement Variables *Note*. Solid lines indicate statistically significant associations. Dotted lines indicate insignificant relationships. *p < .05. **p < .01.

Linear regression and multiple linear regression analyses were used to build predictive models between our variables of interest (Figure 1). To establish a measure of validity, we first checked our results for established age-crime relationships (Rocque et al., 2015; Shulman et al., 2013; U.S. Department of Justice, 2018). Age was a significant predictor of adult legal involvement, β = .27, *t*(110) = 2.95, *p* = .004, accounting for an acceptable proportion of variance therein, *R^2^* = .07, *F*(1, 110) = 8.67, *p* = .004. This offers some evidence that our results are in line with known relationships.

#### Predictive Relationships Between Revised Causal Dimensions Scale Elements

External control significantly predicts personal control, β = -.32, *t*(110) = -3.49, *p* = .001, and accounts for a modest proportion of the variance, *R^2^* = .10, *F*(1, 110) = 12.18, *p* = .001. Additionally, external control is significantly predictive of locus of causality, β = -.31, *t*(110) = -3.46, *p* = .001, and predicted a modest amount of variance, *R^2^* = .10, *F*(1, 110) = 11.99, *p* = .001. Personal control is individually predictive of stability, β = -.29, *t*(110) = -3.13, *p* = .002, but accounts for less of the variance compared to external control, *R^2^* = .08, *F*(1, 110) = 9.97, *p* = .002.

#### Predictive Relationships Between Locus of Control Elements and Legal Involvement Variables

Stability significantly predicts juvenile legal involvement, β = .20, *t*(110) = 2.21, *p* = .03, but accounts for a small proportion of the variance, *R^2^* = .04, *F*(1, 110) = 4.64, *p* = .03. Juvenile involvement is significantly predictive of future likelihood, β = .24, *t*(110) = 2.54, *p* = .01, and accounts for a proportion of the variance in likelihood scores, *R^2^* = .06, *F*(1, 110) = 6.47, *p* = .01, suggesting more juvenile involvement leads to viewing future legal involvement as more likely.

A combined model of stability, personal control, and locus of causality is overall a statistically significant predictor of future likelihood (*p* = .004) and accounts for a significant proportion of the variance there, *R^2^* = .12, *F*(1, 110) = 4.66, *p* = .004. Stability and personal control were both significant predictors in this model individually, β = .21, *t*(110) = 2.19, *p* = .03; β = -.22, *t*(110) = -2.18, *p* = .03, with locus of causality approaching significance as an individual predictor, β = .18, *t*(110) = 1.86, *p* = .07. Thus, higher stability and lower personal control scores significantly predict higher future likelihood scores.

Adult legal involvement is significantly predicted by locus of causality, future likelihood, and juvenile legal involvement. A multiple linear regression containing all three predictors was statistically significant (*p* = .000) and accounted for a sizable proportion of variance in adult involvement, *R^2^* = .20, *F*(1, 110) = 9.24, *p* = .000. Locus of causality, β = .20, *t*(110) = 2.32, *p* = .02, future likelihood, β = .26, *t*(110) = 2.98, *p* = .004, and juvenile legal involvement, β = .23, *t*(110) = 2.57, *p* = .01, all provided significant contributions to the model.

## Discussion

A large majority of individuals who become incarcerated are likely to recidivate throughout life. Up to 83% of released prisoners may be re-arrested or re-incarcerated within three years of release (U.S. Department of Justice, 2018). This problem is partially reflected in our correlation data, with a statistically significant positive correlation between both lifetime and adult legal involvement when compared to age, suggesting that legal involvement increases throughout life for those who end up involved in the system.

Of course, the age-crime relationship tells us that legal involvement increases throughout life as more opportunity for criminality presents itself (Rocque et al., 2015). This relationship is precisely why our correlations are informative. Our results demonstrate that individuals are continuing to capitalize on those opportunities for criminality, rather than opting for non-criminal behaviors upon release from detention.

Literature both explores and acknowledging the socioeconomic stressors such as poverty, violence, addiction, and social contagion that influence this recidivism (Abrams & Snyder, 2010; Dmitrieva et al., 2012; Halliday & Graham, 2000; Han et al., 2001; Lodewijks et al., 2010; Nowicki et al., 2018; Passini, 2012; Richaud, 2013). These are important factors that merit investigation independently beyond the scope of this research. Shulman et al. (2013) demonstrate a factor of independence from these socioeconomic factors in some of the data related to recidivism. This is important for our purposes in that it suggests locus of control can be related to recidivism without the need to use sociocultural variables as a mitigating step.

The established predictive nature of the age-crime relationship in combination with the predictive nature of our results concerning elements of locus of control and criminality is particularly noteworthy. Given that locus of control is an established interaction factor with inmate rehabilitation, measuring an offender’s locus of control and treating it as a predictor of future criminality may help identify those inmates with a higher priority need for intervention (Asberg & Renk, 2014; Morgan & Flora, 2002; Page & Scalora, 2004; van der Helm et al., 2009).

### Hypothesis Evaluation

Our original hypothesis was only partially correct. The predications involving belief in the future likelihood of legal involvement were fully supported by the data. We accurately predicted a positive correlation between legal involvement and beliefs concerning future likelihood. Further, the correlations between future likelihood and *both* stability and personal control suggest an externalizing locus of control related to belief in future likelihood.

The statistically significant path model in Figure 1 illustrates that locus of control may be predictive of increased criminality. Our original hypothesis predicted that this relationship would exist in the opposite direction. While not in support of our original hypothesis, this result is in line with literature that indicated offenders have many opportunities to develop external locus of control prior to their incarceration based on stressful environmental factors (Abrams & Snyder, 2010; Halliday & Graham, 2000; Nowicki et al., 2018; Passini, 2012).

The correlation between adult legal involvement and locus of causality was positive, indicating that more legal involvement reflected viewing the involvement as an aspect of personal quality (an *internal* locus of control trait). It is important to remember that the prompting scenario for the Revised Causal Dimension Scale scores asked participants to describe the overall cause for their legal involvement. This means that stability scores are reflective of how stable (i.e., unchanging) they believe that cause to be. Thus, results suggest that the less changeable one perceives the causes of legal involvement to be, the more likely they will be to believe future involvement will occur.

The positive correlation between stability and both juvenile involvement and future likelihood is also noteworthy. It suggests that individuals with greater juvenile involvement and those viewing future involvement as more likely display proportionate increases in stability scores, suggesting they view the causes of their legal involvement as more stable. Together, these results point to a relationship between legal involvement, locus of causality, stability, and future likelihood.

In an unexpected finding contradictory to our hypothesis, we identified a positive correlation between locus of causality and both adult and lifetime legal involvement. This suggests that those individuals with a greater number of legal involvement occurrences in adulthood are displaying proportionately higher locus of causality scores, and thus more internal locus of control traits.

McAuley et al. (1992) noted a related positive correlation between locus of causality scores and stability scores during the development of the Revised Causal Dimension Scale. This may be because causes viewed as a part of oneself are generally viewed as more stable. In the context of legal involvement, a stable, unchanging cause reflected more external locus of control dimensions. However, viewing the cause as reflecting an aspect of the self rather than an aspect of the situation, as was common among participants, is ultimately reflective of internal locus of control dimensionality. Thus, we note that an increase in this variable appears to be more predictive of recidivism compared to a decrease when it specifically concerns causes for legal involvement.

### Study Limitations

External control was not significantly correlated with any legal involvement variables, though it was negatively correlated with personal control. This may be due to several factors. Take, for example, a personal control question in the scale asking whether the cause is something “you can regulate” or “you cannot regulate” versus an external control question asking if the cause is something “other people can regulate” or “other people cannot regulate” (McAuley et al., 1992). Aligning a certain way on one of those scales does not necessarily mean that the other is not also true. Combined, these factors may be producing the effect we saw in our data. This is an unfortunate limitation in our findings. It must therefore be noted as a potential weakness in this study model that future research on this topic may be able to mitigate.

We did not record the gender of participants, and therefore were unable to include it as a variable in analysis. This was an unfortunate requirement of the IRB which approved the study. We remain unclear regarding the necessity of this requirement, given that the role of an IRB is to assess the risk involved in research with human subjects. Women may have different pathways to criminality compared to men, and this may affect their locus of control in relation to the explored variables. The current study, unfortunately, may be masking these important relationships with the omission of gender data. This issue will be an important one to correct in future studies of this type.

Concerning statistical power, our sample size met requirements for at least a .85 power factor concerning linear regression analysis but fell short of the .85 power factor mark concerning Parson’s *r* correlational analysis. It must be acknowledged that linear regression is dependent on the initial correlation between variables. Therefore, it is notable that the small effect size of our Pearson *r* correlations may have weakened any conclusions here.

Additionally, it remains important to acknowledge that the self-report data measurement method of this research is a notable weakness in terms of objective data accuracy. This is particularly true regarding reported juvenile legal involvement that may have been years behind the offender at the time of participation. We were not permitted to access records for the purposes of cross checking any self-reported information, as county jail inmates may be pre-trial, resulting in the protection of their personal records. Conducting this research in prisons, where individuals have already been sentenced, or in a location with more accessible criminal records may be a partial solution in future studies.

### Conclusions

Together, the correlations and regression models suggest that legal involvement increases with age and that factors of locus of control may predict increased legal involvement and other factors related to recidivism. Results suggest a multifaceted relationship between locus of control and recidivism. The hypothesized direction of the relationship was incorrect, yet a predictive relationship still exists. Together, the results of the regression analyses suggest that (a) identifying measurable scores on locus of control would allow for a prediction of how likely an individual believes future legal involvement is and (b) may allow for predictive identification of those at high risk for recidivism, which we defined as increased adult legal involvement.

The significance of our data rests in the suggestion that high-recidivism individuals may already have elements of external locus of control when they enter the system. This may fuel a continuation in criminality. Further, our correlations suggest that offenders view criminality as a personal attribute, yet still view the related life circumstances as unchangeable. Linear regression analysis also provides evidence to support this latter portion, illustrating that such control-based orientations may be predictive of increased legal involvement in adulthood. Together, these results suggest that addressing control orientations centered on life circumstances may be an effective strategy for offender rehabilitation. Further research is necessary to properly evaluate this in a treatment-oriented setting.

It is also possible that identifying those individuals with high stability and low personal control orientations related to these legal causes may serve as the first indicator to high recidivism risk. If these patterns could be identified while the offender still has relatively low legal involvement, they may be more likely to break the criminal cycle following control orientation-based treatment. Further research is needed to properly evaluate the efficacy of these speculations. Nonetheless, our results provide an important foundation to these future rehabilitative directions.
